# Immunity, safety and protection of an Adenovirus 5 prime - Modified Vaccinia virus Ankara boost subunit vaccine against *Mycobacterium avium* subspecies *paratuberculosis* infection in calves

**DOI:** 10.1186/s13567-014-0112-9

**Published:** 2014-10-29

**Authors:** Tim J Bull, Christina Vrettou, Richard Linedale, Catherine McGuinnes, Sam Strain, Jim McNair, Sarah C Gilbert, Jayne C Hope

**Affiliations:** Institute of Infection and Immunity, St. George’s University of London, Cranmer Terrace, London, SW17 0RE UK; The Roslin Institute, University of Edinburgh, Easter Bush, Midlothian, EH25 9RG UK; Animal Health and Welfare Northern Ireland, 97 Moy Road, Dungannon, Co. Tyrone, BT71 7DX Northern Ireland UK; Agri-Food and Biosciences Institute, Stoney Road, Stormont, Belfast, BT4 3SD Northern Ireland UK; The Jenner Institute, University of Oxford, Old Road Campus Research Building, Oxford, OX3 7DQ UK

## Abstract

Vaccination is the most cost effective control measure for Johne’s disease caused by *Mycobacterium avium* subspecies *paratuberculosis* (MAP) but currently available whole cell killed formulations have limited efficacy and are incompatible with the diagnosis of bovine tuberculosis by tuberculin skin test. We have evaluated the utility of a viral delivery regimen of non-replicative human Adenovirus 5 and Modified Vaccinia virus Ankara recombinant for early entry MAP specific antigens (HAV) to show protection against challenge in a calf model and extensively screened for differential immunological markers associated with protection. We have shown that HAV vaccination was well tolerated, could be detected using a differentiation of infected and vaccinated animals (DIVA) test, showed no cross-reactivity with tuberculin and provided a degree of protection against challenge evidenced by a lack of faecal shedding in vaccinated animals that persisted throughout the 7 month infection period. Calves given HAV vaccination had significant priming and boosting of MAP derived antigen (PPD-J) specific CD4^+^, CD8^+^ IFN-γ producing T-cell populations and, upon challenge, developed early specific Th17 related immune responses, enhanced IFN-γ responses and retained a high MAP killing capacity in blood. During later phases post MAP challenge, PPD-J antigen specific IFN-γ and Th17 responses in HAV vaccinated animals corresponded with improvements in peripheral bacteraemia. By contrast a lack of IFN-γ, induction of FoxP3+ T cells and increased IL-1β and IL-10 secretion were indicative of progressive infection in Sham vaccinated animals. We conclude that HAV vaccination shows excellent promise as a new tool for improving control of MAP infection in cattle.

**Electronic supplementary material:**

The online version of this article (doi:10.1186/s13567-014-0112-9) contains supplementary material, which is available to authorized users.

## Introduction

*Mycobacterium avium* subspecies *paratuberculosis* (MAP) is the causative agent of Johne’s disease (JD), a chronic granulomatous inflammation of the intestines primarily in ruminants [1] and which has been linked to Crohn’s disease in humans [2]. The increasing prevalence of MAP infection in cattle, the associated economic losses and zoonotic potential indicate the need for an effective MAP vaccine as a sustainable and economically viable solution for disease control [3]. Whole cell killed MAP vaccines can improve milk productivity [4] reduce the incidence of clinical JD [5] and faecal abundance of MAP [6], however the proportion of animals harbouring and excreting MAP remain similar [7]. Attempts to provide live attenuated whole cell vaccines have mostly been ineffective [3]. Although some have shown promise in goats [8], none have been able to eliminate faecal excretion. This is an important pre-requisite, however recent modelling studies indicate that to achieve effective MAP control in cattle in the shortest time period, priority should be given to vaccines that are able to reduce susceptibility to MAP infection [9]. In contrast, vaccines targeted at only reducing clinical cases and decreasing but not eliminating MAP shedding would not be economically beneficial to dairy producers when compared with an alternative non-vaccine control, particularly when herds were highly infected with MAP [10]. In the study reported here we have assessed protective capacity as an ability of vaccination to reduce bacterial numbers in peripheral blood, gut tissues and in faeces as this is likely to impact significantly on disease progression and transmission.

Importantly whole cell MAP vaccines contain generic mycobacterial cell wall antigens cross-reactive with tuberculin [11] used in diagnostic testing for bovine tuberculosis caused by *Mycobacterium bovis*. In addition animals vaccinated with whole cell MAP vaccines cannot be differentiated, using current diagnostic tests, from MAP infected animals and there is therefore a need for any new MAP vaccine to have a Differentiation of Infected from Vaccinated Animals (DIVA) test capability [12]. Eradication programs are currently impossible whilst inter-animal spread and seeding into environmental or wildlife reservoirs remains high. Therefore whole cell vaccines have limited value in cattle control programmes given their limited efficacy and their interference with current bovine TB diagnostics, particularly in areas with ongoing endemic bovine tuberculosis [13].

Of increasing importance is the understanding that MAP disease involves an insidious onset of dysregulatory immune mechanisms that need to be normalised or prevented from accumulating if protection is to be achieved. Comparisons with studies in other mycobacterial diseases suggest lasting effective immunity would require combinations of humoral and mucosal immunity along with priming and maintenance of pathogen specific Th1 responses [14]. Development of an effective vaccine thus requires rational, focused design using novel delivery methods able to circumvent dysregulated antigen priming invoked during MAP persistence whilst specifically targeting and enhancing the crucial immunological processes able to arrest pathogenesis.

The ability of viral vectored vaccines to positively address these processes has already shown promise in other mycobacterial diseases including cattle [15-17]. Viral delivery provides the advantages of high antigen delivery to antigen presenting cells [18], increased antigen specific CD4^+^ and CD8^+^ responses [19,20] and maintenance of IFN-γ release driving increased macrophage activation and killing capacity [21]. Prime-boost vaccination with MVA-Ag85A induced altered Th1/Th17 related responses [22] that were shown to correlate with the induction of protective immunity [17].

We have previously demonstrated that a priming vaccination with non-replicative Adenovirus 5 followed by boosting with Modified Vaccinia virus Ankara delivery vectors expressing a fusion of critical epitopes from four intracellular phase codon optimised MAP antigens (HAV) was able to confer both therapeutic and prophylactic protection against MAP challenge in mice [23]. In this study we evaluate the same prime boost strategy in cattle and investigate immunological responses associated with protection. We show that HAV vaccination was well tolerated, could be detected by DIVA testing, did not cross react with the tuberculin test and provided a high degree of protection against challenge evidenced by a lack of faecal shedding that persisted throughout the 38-week test period.

## Materials and methods

### Vaccine construction and manufacture

HAV vaccine comprised non replicative human adenovirus serotype 5 (Ad5) and Modified Vaccinia virus Ankara (MVA) expressing a 838aa polypeptide fusion of regions sourced from four non-essential, non-toxigenic, immunogenic, early intracellularly expressed MAP proteins with no significant BLASTP homologies to either mammalian or known tuberculosis PPD proteins [11]. Vaccine design and production including extensive codon optimisation and addition of expression enhancement features were as described previously [23]. Vaccine doses were derived from a single batch preparation manufactured at the Viral Vector Core Facility, Jenner Institute, Oxford, UK from seed stocks using specific pathogen free CEF and T-Rex 293A cell lines for MVA and Ad5 respectively in certified pathogen free media. Ad5 and MVA vectors expressing GFP in place of the HAV polypeptide were manufactured in parallel to a similar viral titre and used for Sham vaccination.

### Preparation of MAP challenge inoculum

A strain of MAP (R0808) isolated from a cow with JD was inoculated into modified liquid Middlebrooks 7H9 medium [24] and grown at 37 °C with gentle agitation to an OD_600_ of 1.8. The culture was extensively passed through a 20G needle and any remaining large clumps were allowed to settle for 5 min. The upper suspension was then separated and adjusted to an OD_600_ of 1.0 with sterile PBS then aliquoted in 5 mL doses. qPCR using MAP specific primers (see below) estimated that each dose contained 5 × 10^8^ MAP genome equivalents of MAP organisms. Prior to dosing aliquots were centrifuged at 3500 × *g* for 10 min and the pellet resuspended with a syringe into 20 mL of PBS.

### Vaccination, challenge and sampling regimen

Calves were selected from herds with an absence of tuberculin skin test reactivity over the previous 10 years, and without bovine tuberculosis disclosed at abattoir over the same period. These same herds were selected on the basis that increases in skin thickness following injection of PPD-A were minimal, below 4 mm, indirectly indicating an absence of immune responses to MAP. Eight week old male Holstein Friesian calves in two groups of six were held under regulated category containment at AFBINI, Stormont, Northern Ireland and vaccinated intradermally into the neck region with 1 mL of either Ad5.HAV (10^9^ vp/mL) at week -11 and boosted with MVA.HAV (10^9^ pfu/mL) at week -5 (HAV) or vaccinated with Ad5 (10^9^ vp/mL) and MVA (10^9^ pfu / mL) vectors expressing GFP in the same regime (Sham). Five weeks after boosting (week 0) both vaccinated groups were challenged on two consecutive days with an oral dose of 5 × 10^8^ MAP R0808 mixed into PBS.

Faecal and blood samples were taken immediately prior and 1 week post- prime and boost vaccinations and post-challenge, then monthly over the 38 week post challenge study period. Whole bloods were processed for IFN-γ release assays. PBMC fractions were aliquoted and processed separately for MAP killing assays, MAP presence using a 2 week pre-liquid culture followed by subculture for MAP colonies on solid slopes and direct differential lysis DNA extraction for MAP by nested PCR, cytokine expression or presence of HAV transgene using cDNA extractions followed by specific PCR, cytokine release assays by ELISA and cell population analysis using flow cytometry. Faecal samples were processed for MAP presence by 2 week pre-liquid culture followed by dividing for subculture on solid slopes and differential lysis DNA extraction for MAP by nested PCR. Direct DNA extraction was also performed separately on faecal samples for HAV transgene PCR testing. Animals were euthanized at 38 weeks post challenge and samples from mesenteric lymph nodes, duodenum, spleen, ileum and jejunum taken at necropsy. Tissue samples were processed for MAP load by direct differential mycobacterial DNA extraction followed by qPCR, for cytokine expression and HAV transgene presence by PCR from cDNA preparations and cell population analysis using flow cytometry.

All animal husbandry and procedures were carried out by trained and experienced animal care workers under the direction of a senior Named Veterinary Surgeon and in compliance with the UK Home Office Regulations including the Animals (Scientific Procedures) Act 1986. The study was authorized by the local Ethical Review Committee at AFBINI, in compliance with national guidelines and EU regulations for projects using animals for research purposes.

### Tissue preparation

All tissue samples were dissected at necropsy and, for MAP culture and DNA isolation, were initially stored/transported for up to two days at RT in 1 mL RPMI1640 (Sigma, Gillingham, UK) plus 10% FBS and 100 μg/mL ampicillin. For cell isolation for flow cytometry and RNA isolation tissues were processed immediately following retrieval. Fat was removed from the tissue and mucosal tissue (if present) was scraped, washed with PBS, diced using sterile scalpels and then weighed. Lymph nodes were gently dissociated using a scalpel and a single cell suspension was obtained by filtration through a 70 μm cell strainer (Becton Dickinson, Oxford, UK). For MAP culture and DNA PCR, samples were digested overnight with slow agitation at 37 °C in 1 mL Pen/Strep free digest buffer (0.15 mM NaCl, 0.3 mM CaCl_2_, 1 mg/mL collagenase B, 1 mg/mL trypsin (Sigma)) then pelleted in a microfuge at 16 000 × *g* for 5 min. For flow cytometric analysis and host RNA extraction, ileum and ileocecal valve tissue was digested for 80 min at 37 °C in digestion medium (RPMI1640, 1% FBS, 25 μg/mL gentamicin, 100 U/mL Pen/Strep, 75 U/mL collagenase B (Sigma), 20 μg/mL Dispase I (Roche, Welwyn Garden City, UK)). The cells were then washed gently in PBS. For flow cytometric analysis tissue cells were fixed with 1% paraformaldehyde and stored at 4 °C. For RNA expression analysis preparations were resuspended in 1 mL of RLT Plus buffer (Qiagen, Manchester, UK) containing 1 μL/mL β-mercaptoethanol prior to RNA extraction.

### Isolation and stimulation of PBMC

PBMC were isolated by density gradient centrifugation (Histopaque 1083 (Sigma), resuspended in tissue culture medium ([TCM]; RPMI +10% foetal calf serum, 5 × 10^−5^ M β-mercaptoethanol, 50 μg/mL gentamicin). Cell concentrations were estimated using a haemocytometer, adjusted to 1 × 10^7^ cells/mL then aliquoted (5 × 10^6^ cells) for MAP and HAV transgene PCR or stimulated with either 10 μg/mL PPD-J (a kind gift from D Bakker, CVL, Lelystad, Netherlands) or an equal volume of TCM alone as control for 24 h at 37 °C in 5% CO_2_ in air. Stimulated or unstimulated PBMC were then pelleted and supernatants stored at −70 °C prior to cytokine analysis by ELISA. Parallel stimulations were established for the subsequent analysis of cytokine expression by qPCR and multiparametric analysis of cell populations by flow cytometry.

### DNA extraction

Pelleted samples were suspended in 600 μL GTC buffer (4 M Guanidium thiocyanate (Sigma), 10 mM TrisHCl, pH 8.0, 1 mM EDTA), transferred to a 1.5 mL lysing matrix B ribolyser tube (MP Biomedicals, Leicester, UK) mixed and lysed overnight at 4 °C. DNA extraction included mechanical disruption in a FastPrep-24 ribolyser (MP Biomedicals) at 6500 rpm for 45 s followed by standard extraction using phenol/chloroform, chloroform/isoamyl alcohol and precipitation overnight at −20 °C with 7.5 M ammonium acetate in ethanol [25].

### MAP specific PCR

qPCR of DNA extracted from tissue samples was performed as previously described with minor adjustments [26]. Briefly, reactions comprised 2 μL DNA sample, 12.5 μL Power SYBR green mastermix (Applied Biosystems, Paisley, UK), 2 pMoles primer pair (AV1: ATGTGGTTGCTGTGTTGGATGG, AV2: CCGCCGCAATCAACTCCAG), made to 25 μL with RNAse free water. PCR cycling used 95 °C: 15 min (1 cycle); at 95 °C: 30 s, 58 °C: 1 min, 72 °C: 1 min (40 cycles) with data collection at 76 °C (10 s) using a CFX96 qPCR cycler (BioRad, Hemel Hempstead, UK). Sample copy numbers were estimated from an averaged value of three qPCR’s on each sample using a dilution curve of a control total genomic DNA MAP K-10 stock preparation serially diluted 10 fold to contain between 1 × 10^2^-10^8^ genome equivalents. Nested MAP specific PCR of DNA extracted from PBMC and liquid pre-culture faecal sample preparations was performed as previously described [25].

### HAV transgene specific PCR

Blood and faecal samples taken 1 week prior to prime vaccination then at intervals post vaccination (week -10, -4, 0, 6 and 33) and spleen samples taken post mortem (week 38) from each vaccinated animal were screened for the presence of the HAV transgene. DNA was extracted from PBMC and tissue samples as described above. Faecal samples (200 mg) were processed using a QIAamp Stool DNA extraction kit (Qiagen) using a standard protocol optimised for viral DNA extraction [27]. DNA extracted from MVA.HAV cultured in CEF cells (48 h: MOI 50:1) using either blood or faecal extraction method were used as positive reagent/lysis controls. PCR sensitivity was estimated at 20-50 copies (positive in > 80% replicates) by dilution curves of a reference DNA standard stock comprising a plasmid containing one copy of the HAV transgene construct.

### RNA extraction and cDNA generation

Total RNA was isolated from PBMC stored in 1 mL of RLT Plus buffer (Qiagen) containing 1 μL/mL β-mercaptoethanol using the ALLPrep DNA/RNA mini kit (Qiagen) and the robotic workstation for the automated isolation of DNA and RNA, Qiacube (Qiagen), according to manufacturer’s instructions. First strand cDNA was synthesised from 250 ng mRNA sample aliquots using standard reverse transcription reaction buffer (10 mM dNTPs (Bioline, London, UK), 500 μg/mL oligo(dT)_15_ primers (Promega, Southampton, UK), 5 × RT Buffer, 0.1 M DTT and Superscript II Reverse Transcriptase (Invitrogen, Paisley, UK)) denatured at 65 °C for 10 min and incubated at 42 °C for 50 min.

### cDNA qPCR for IL-17, IL-22 and IL-23 expression

IL-17, IL-22, IL-23 and the reference gene GAPDH were amplified by qPCR using the LightCycler 480 DNA SYBR Green I Master on the LightCycler 480 qPCR machine (Roche). The primers and conditions used were: IL-17A: FW: TAACCGGAGCACAAACTCCAGA ; RV: GGTGGAGAGTCCAAGGTGAGGT; 95 °C: 5 min (1 cycle), then 95 °C: 20 s, 62 °C: 20 s, 72 °C: 30 s (45 cycles): IL-22: FW: CCGCTGGCTGCCTCCTT; RV: AGGGCTCCTGGAAGTCGGA ; 95 °C: 5 min (1 cycle), then 95 °C: 20 s, 60 °C: 20 s, 72 °C: 30 s (40 cycles): IL-23: FW: ACCAATGGGACATGTGGATCTAC; RV: AGGGCTTGGAGTCTGCTCAGTT: 95 °C: 5 min (1 cycle), then 95 °C: 20 s, 60 °C: 20 s, 72 °C: 30 s (45 cycles): GAPDH: FW: GATGCTGGTGCTGAGTATGTAGTG RV: ATCCACAACAGACACGTTGGGAG 95 °C: 5 min (1 cycle), then 95 °C: 20 s, 60 °C: 20 s, 72 °C: 45 s (40 cycles).

All reactions were run in duplicate in a final volume of 20 μL. Relative gene quantities were calculated using LightCycler480 1.5.0 software by comparing each sample with a serial dilution of standard PCR purified products in the same run. Concentrations of genes of interest were then calculated from standard curves in arbitrary units. Antigen-specific gene induction was calculated according to the method described by Pfaffl [28] from values of each target gene normalized to the reference gene (ref; GAPDH) for each sample. Briefly, values were calculated according to the following:

$$ \mathrm{Ratio} = \frac{{\left({\mathrm{E}}_{\mathrm{target}}\right)}^{\Delta \mathrm{C}\mathrm{p}\ \mathrm{target}\ \left(\mathrm{control}\hbox{-} \mathrm{sample}\right)}}{{\left({\mathrm{E}}_{\mathrm{ref}}\right)}^{\Delta \mathrm{C}\mathrm{p}\ \mathrm{r}\mathrm{e}\mathrm{f}\ \left(\mathrm{control}\hbox{-} \mathrm{sample}\right)}} $$

Where E is efficiency calculated by the standard curve for each gene and ΔCp is the difference calculated by the Lightcycler of the samples treated with media alone minus the samples treated with PPD-J.

### MAP culture

Samples were decontaminated and extracted following previously described recommended guidelines [29] then cultured on liquid Middlebrooks 7H9 medium [24] at 37 °C for 2 weeks, then plated onto the same medium (with added agar) and incubated for up to 12 weeks or until colonies appeared. PBMC samples (2 × 10^6^ cells) were added to 5 mL of sterile distilled water and lysed for 30 min then centrifuged for 15 min at 3500 × *g* and the pellet resuspended in 1 mL modified liquid Middlebrooks 7H9 medium and cultured as faecal samples. Colony identity was confirmed with MAP specific PCR as above and representative isolates from the initial inoculum and a final faecal sample also subjected to MIRU typing.

### MAP killing assay

Whole blood (15 mL) in EDTA tubes was mixed with 15 mL PBS and centrifuged at room temperature onto 15 mL Histopaque 1083 (Sigma) for 1200 × *g* for 1 h with no brake applied. Buffy coats were pipetted off and washed once in PBS. Cells were diluted to 1 × 10^7^/mL in RPMI medium (RPMI1640, 10% FCS, 50 μg/mL Hygromycin B) then plated into 96 well flat bottom tissue culture plates at 4 × 10^5^ cells per well in quadruplicate. Two duplicates were activated with 30 ng/mL bovine IFN-γ (Fisher Scientific, Loughborough, UK) then incubated at 37 °C in 5% CO_2_ overnight to attach. Media was exchanged with 200 μL of RPMI containing 8 × 10^5^ luminescent MAP 19698 L [30] and incubated for 5 days in 5% CO_2_, changing media at 3 days. Cells were washed once in PBS then lysed in 200 μL 0.4% SDS final in PBS and read immediately in an injector Luminometer GloMax 20/20 (Promega) set at 1 s delay, using 1% v/v decanal (Sigma) as substrate. Relative killing values were calculated as the percentage of luminosity lost from an average of both IFN-γ activated and non-activated infected cell cultures relative to an RPMI only infection control set of wells.

### Whole blood IFN-γ release assay

Whole blood IFN-γ release assay was performed as previously described [31]. Briefly heparinised whole blood was stimulated within one hour of sampling with either PBS (control), avian-purified protein derivatives (PPD-A) at 4 μg/mL final concentration (Veterinary Laboratories Agency, Guildford, UK), bovine-purified protein derivative (PPD-B) at 8 μg/mL final concentration (Veterinary Laboratories Agency, UK), and Johnin-purified protein derivative (PPD-J) at 4 μg/mL final concentration (Central Veterinary Institute, Copenhagen,Denmark), pokeweed mitogen (Sigma) (positive control) and a set of peptides (Pool J) spanning MAPK_1565 (C-term) plus MAPK_2533 (N-term) region of the HAV transcript (GKRHTQAVLALARRR; QAVLALARRRLNVLW; LARRRLNVLWAMLRD; LNVLWAMLRDHAVYH; AMLRDHAVYHPATTT; HAVYHPATTTAAARL; SIVGQTYREVEVVLD; TYREVEVVLVDGGST; EVVLVDGGSTDRTLD; DGGSTDRTLDIANSF) at a final concentration of 2 μg/mL each [23]. After 24 h, plasma was tested in duplicate by Bovigam ELISA (Prionics, Lelystad, Netherlands) for the release of bovine IFN-γ. Values are expressed as a Net OD (OD of antigen stimulated sample minus OD of negative control).

### ELISA for IL-1β and IL-10 expression

The supernatants were assessed for the presence of IL-1β using a bovine IL-1β kit (Thermofisher, Loughborough, UK) and for IL-10 as previously described [32]. The concentration of IL-1β is expressed as pg/mL and for IL-10 as biological units (BU)/mL relative to a standard curve. For IL-10 the standard preparation was CHO cell expressed IL-10 (a kind gift from G Entrican, Moredun Research Institute, Edinburgh). Each sample assayed was measured in duplicate by ELISA; the variability between samples was less than 5%.

### Multi-colour immunofluorescent labelling

PBMC stimulated for 24 h with PPD-J or TCM were harvested and subjected to multi-parametric staining protocols. Unless indicated all primary monoclonal antibodies were from AbD-Serotec (Kidlington, UK) and secondary antibodies were: goat anti-mouse IgG1-alexa-fluor 647 (Life Technologies, Paisley, UK), goat anti-mouse IgG2a-PECy7 (Abcam, Cambridge, UK), goat anti-mouse IgG2b-RPE and goat anti-mouse IgG3-FITC (Cambridge BioScience, Cambridge, UK). All antibodies were used at predetermined optimal concentrations. The fluorescence without the presence of primary mAb was used as a control for analysis. Four colour flow cytometry was utilised to define cell subsets. T lymphocyte subsets were detected using mAbs specific for bovine CD4 (CC30, IgG1 or CC8, IgG2a), CD8 (CC58, IgG1 or CC63, IgG2a), the WC1 γδ TCR (CC15, IgG2a) or pan-γδ TCR (GB21a, IgG2b; VMRD, Pullman, USA). The expression of CD25 (IL-A111, IgG1) and CD45RO (IL-A116, IgG3) was determined on subsets of T cells. Intracellular expression of FoxP3 was determined within cells that were fixed with 1% paraformaldehyde and permeabilised (BD FACSPerm) using mAb Fox5A (anti-bovine Foxp3, IgG1 [33]; a gift from Professor WC Davis, Washington State University, USA). For intracellular staining of IFN-γ, cells were pre-incubated with TCM with and without PPD-J supplemented with PMA, ionomycin and brefeldin A (Sigma), then fixed and permeabilised as described above. Cytokine expression was determined using anti-bovine IFN-γ (CC330, IgG1). Flow cytometric analysis was conducted using the FACSCalibur (for intracellular IFN-γ expression) or the LSR II Fortessa (Becton Dickinson) and a minimum of 10 000 events were collected. Flow cytometric data was analysed using FlowJo software (v.7.6.5).

### Statistical analysis

Group sizes were calculated using G*Power program (v.3.15) based upon standard deviations from a similar study [31] calculated at alpha significance of 0.05 to derive an expected 90% power probability using a two tailed t-test. Statistical analyses were calculated using a standard statistics package software (GraphPad Prism v.6.04, La Jolla, USA) or in SAS using a mixed model for repeated measures analysis.

## Results

### Vaccination and general condition of animals

Calves were vaccinated at week -11 with Ad5-HAV, boosted at week -5 with MVA-HAV (HAV vaccinated group) then challenged orally with MAP at week 0. A second group were vaccinated and challenged under the same regime but with Ad5-GFP and MVA-GFP controls (Sham vaccinated group). Vaccine preparations gave no adverse reactions at any time during the experiment. No significant swelling or induration was observed at any of the vaccination sites. PCR for HAV transgene specific DNA, carried out on blood and faecal samples taken at intervals throughout the experiment and spleen tissue at necropsy, was uniformly negative demonstrating that no vaccine was shed from the animals (data not shown). One calf in the Sham vaccinated group developed an unrelated illness (determined by post-mortem examination as septicaemia related to a navel infection) 3 weeks post vaccination and was euthanized. All other calves appeared healthy throughout the experiment. There was no significant decrease in the final body weights of the groups (data not shown). It became evident during data analysis that there were distinct phases in several parameters that differed between groups. We therefore report these findings in relation to each of these sequential phases.

### Pre-challenge

**Figure 1:**
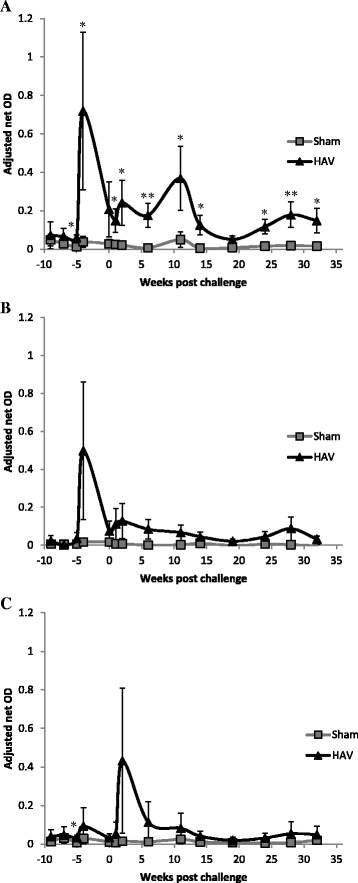
**Whole blood PPD-J stimulated IFN-**γ **release assay**. IFN-γ release from **A**. PPD-J, **B**. PPD-A, **C**. PPD-B stimulated whole blood taken from HAV vaccinated (black triangles) and Sham vaccinated (grey squares) calves including samples taken week -11, immediately prior prime vaccination; week -5, immediately prior boost vaccination; week 0, immediately prior to MAP challenge and up to 33 weeks post challenge. OD values are adjusted to internal controls to remove assay variation between runs. Significance indicated as *P <0.05, **P <0.01.

**Figure 2:**
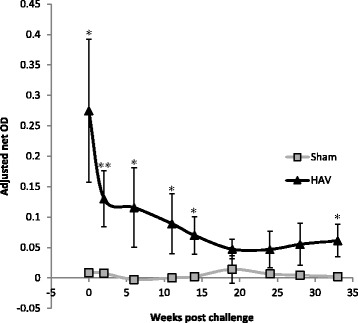
**DIVA testing.** IFN-γ release from HAV specific peptide pool stimulated whole blood taken from HAV vaccinated (triangles) and Sham vaccinated (squares) calves between week 0, immediately prior to MAP challenge and 33 weeks post challenge. OD values are adjusted to internal controls to remove assay variation between runs. Significance indicated as * *P < *0.05.

All of the calves in the HAV vaccinated group, but not the Sham vaccinated group responded to vaccination with an increase in MAP (PPD-J) specific IFN-γ release (Figure 1A). A significant increase (*P < *0.05) in the level of PPD-J specific IFN-γ released from stimulated whole blood was evident at one week post-MVA-HAV boosting and this remained significantly elevated throughout all but one testing month in the experimental period (*P < *0.05). By contrast no PPD-J specific IFN-γ was detected following Sham vaccination. Increases in avium (PPD-A) and bovine (PPD-B) specific IFN-γ release were also evident in the HAV- but not the Sham vaccinated calves immediately post-MVA boost but these did not reach significance and rapidly declined to baseline prior to challenge (Figure 1B and C). In parallel we assessed IFN-γ release by whole blood stimulated with a pool of HAV specific peptides in order to determine whether these could be used to distinguish vaccinated from infected animals (DIVA). In response to HAV peptides we observed a significant increase in IFNγ responses (*P < *0.05) from blood of HAV vaccinated but not Sham vaccinated animals. This remained elevated for the duration of the experiment (Figure 2).

**Figure 3:**
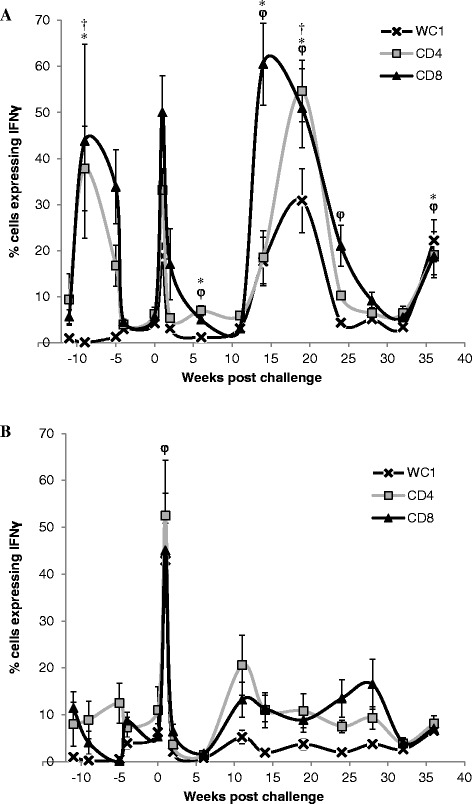
**Intracytoplasmic expression of IFN-**γ **by sub-populations of peripheral T cells in HAV and Sham-vaccinated calves.** Percentage of live CD4^+^ (squares), CD8 ^+^ (triangles) WC1 ^+^ (crosses) expressing IFN-γ after 24 h stimulation with PPD-J from A. HAV vaccinated calves B. Sham vaccinated calves including samples taken week -11, immediately prior prime vaccination; week -5, immediately prior boost vaccination; week 0, immediately prior to MAP challenge and up to 36 weeks post challenge. Significant differences between groups in **A**. and **B**. of *P < *0.05 are indicated as † (CD4^+^), * (CD8 ^+^), φ (WC1 ^+^) for each time point.

In stimulated PBMC we observed differences in PPD-J specific IFN-γ expression by subsets of T lymphocytes from HAV (Figure 3A) and Sham (Figure 3B) vaccinated calves. In Sham vaccinated calves no significant differences in the percentage of cells expressing IFN-γ in response to PPD-J were observed pre-challenge. By contrast, significant differences (*P < *0.05) in PPD-J specific CD4^+^IFN-γ^+^ and CD8^+^IFN-γ^+^ cells were observed pre-challenge in HAV vaccinated calves which peaked 2 weeks (week -9) post Ad5-HAV vaccination. No differences were observed in IFN-γ expression by WC1^+^ γδ TCR^+^ T cell populations.

### Post challenge (1-5 weeks)

**Figure 4:**
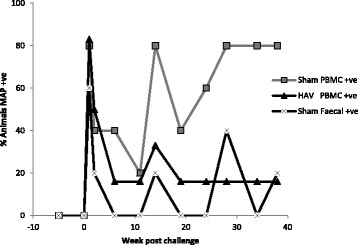
**MAP PCR positivity in blood and faeces of HAV and Sham vaccinated calves.** Percentage of animal samples that were positive for MAP by PCR. MAP was assessed in PBMC from HAV vaccinated calves (triangles), PBMC from Sham vaccinated calves (squares) and faeces from Sham vaccinated calves (crosses). Faeces of HAV vaccinated calves were consistently negative (data not shown) from immediately prior to boost vaccination (week -5) up to 36 weeks post challenge.

**Figure 5:**
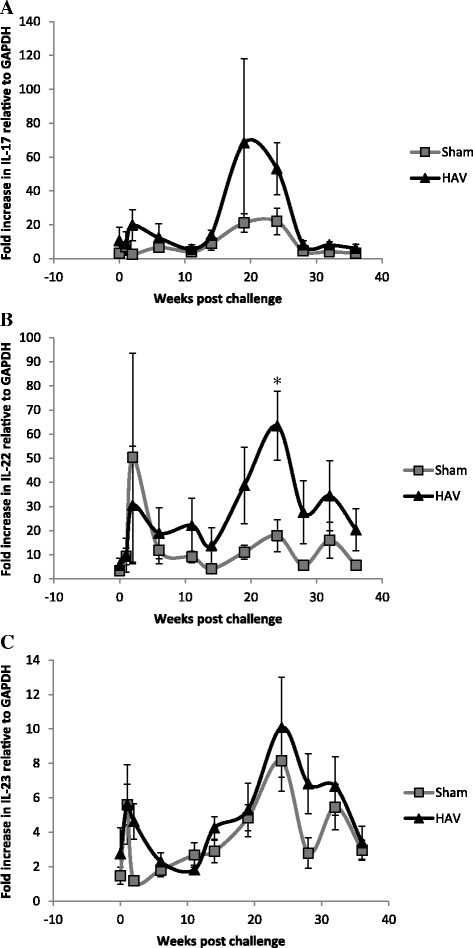
**Expression of antigen specific IL-17, IL-22 and IL-23 by PBMC from HAV- and Sham-vaccinated calves.** Fold increases, relative to GAPDH. in expression of cytokines **A**. IL-17, **B**. IL-22 and **C**. IL-23 measured by qPCR from RNA extracted from 24 h PPD-J stimulated PBMC isolated from HAV vaccinated (triangles) or Sham vaccinated (squares) calves, taken immediately prior to MAP challenge (week 0) up to 36 weeks post challenge. Significance between groups is indicated as * *P < *0.05.

Post oral MAP challenge (week 0) we systematically assessed antigen-specific immune responses in whole blood and isolated PBMC populations, the presence of MAP DNA in blood and faecal samples and macrophage killing efficacies. One week following oral challenge PBMC isolated from 5/6 (83%) HAV vaccinated and from 4/5 (80%) Sham vaccinated calves became MAP PCR positive and 3/5 (60%) Sham vaccinated calves shed MAP in faeces (Figure 4). Faecal cultures for HAV vaccinated calves were negative for MAP throughout the experiment. Two weeks following challenge PBMC from 3/6 (50%) HAV vaccinated and 2/5 (40%) Sham vaccinated calves were positive for MAP by PCR and 1 of the 5 calves (20%) in the Sham vaccinated group was still shedding MAP in the faeces (Figure 4). At week 1 post challenge a transient but significant (relative to week 0) peak in the percentage of PPD-J specific IFN-γ^+^ T cells (CD4^+^, CD8^+^; *P < *0.001) and WC1^+^ subsets (*P < *0.01) was observed in all animals (Figure 3). In whole blood significantly (*P < *0.05) higher levels of PPD-J specific IFN-γ release were also detected in the HAV vaccinated group compared to the Sham vaccinated calves and this remained significantly elevated throughout the course of the experiment (Figure 1). Alongside alterations in T cell populations expressing IFN-γ occurring at this early time point post-MAP challenge, we observed a significant increase in IL-22 (*P < *0.05) and a trend towards increased IL-17 expression in the HAV vaccinated but not the Sham vaccinated animals (Figure 5).

**Figure 6:**
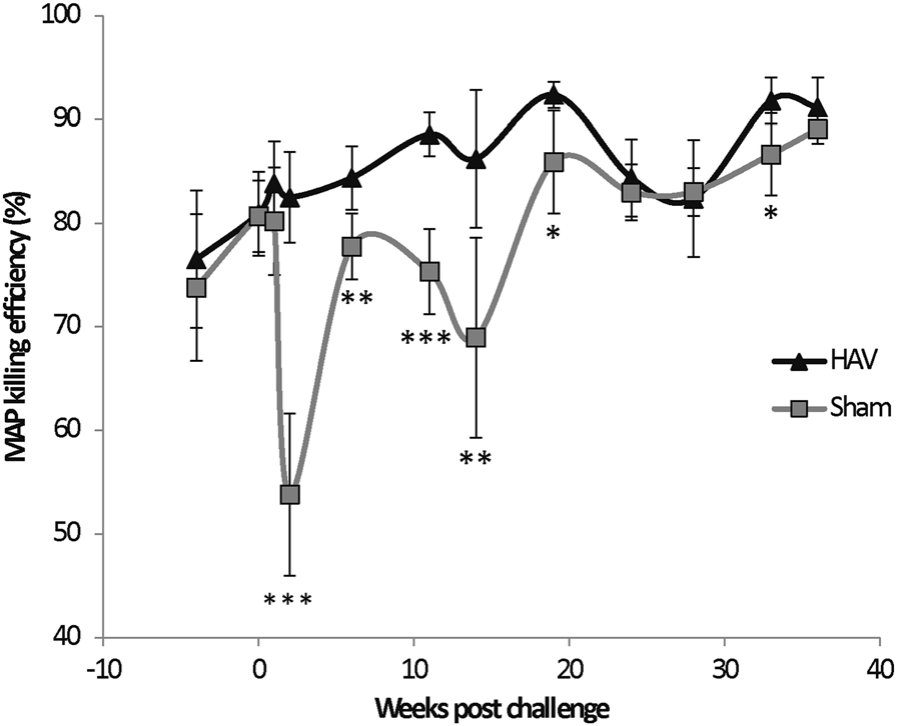
**MAP killing efficiency of macrophage fractions.** Percentage of an initial MAP inoculum killed after infection for 5 days in macrophages isolated from HAV vaccinated (diamonds) or Sham vaccinated (squares) calves taken immediately prior to HAV boost (week -5) up to 36 weeks post challenge. Values given are averages of bovine IFN-γ stimulated and unstimulated preparations performed in duplicate. Significance between groups is indicated as * *P < *0.05, ** *P < *0.01, *** *P < *0.001.

Immediately prior to challenge PBMC isolated from Sham vaccinated calves were equally capable of killing MAP compared to PBMC from HAV vaccinated calves. However, within one week of challenge the efficacy of PBMC fractions to kill MAP dropped dramatically by ~30% in the Sham group whereas the capacity for MAP killing was retained within the HAV animals (Figure 6). This large drop in killing efficacy was of relatively short duration with some recovery of killing capacity by 2 weeks post-challenge but a significant difference (*P < *0.05) between HAV and Sham vaccinated animals remained evident between weeks 1 to 24 post-challenge.

### Post challenge (6-19 weeks)

Significant differences between the HAV and Sham vaccinated groups across a range of parameters were observed between weeks 6 and 14. The MAP killing capacity of PBMC from the Sham vaccinated group remained reduced (Figure 6) and there was an increase in the number of animals with MAP positive PBMC. PBMC from one animal were positive for MAP by PCR in each group at week 11 and this had increased to 4/5 (80%) Sham vaccinated and 2/6 (33%) HAV vaccinated calves positive at 14 weeks (Figure 4).

**Figure 7:**
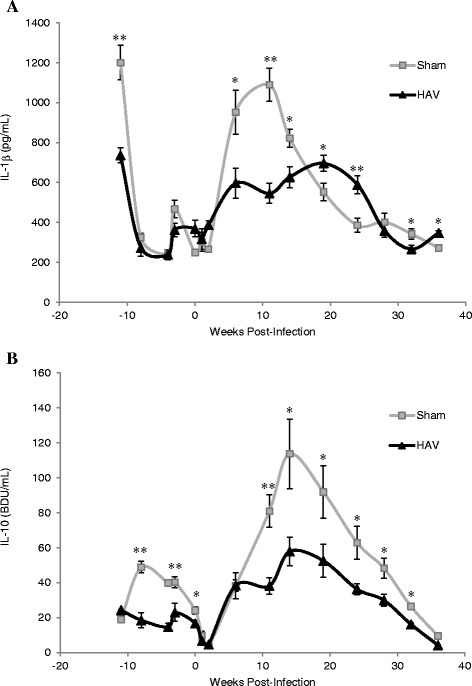
**Secretion of IL-1**β **and IL-10 by PBMC from HAV- and Sham-vaccinated animals.** At the indicated time points post-MAP challenge PBMC were isolated from HAV-vaccinated (triangle) or Sham-vaccinated (grey square) calves and stimulated for 24 h with PPD-J or left unstimulated (control). Supernatants were assessed for the presence of **A**. IL-1β and **B**. IL-10 in triplicate by ELISA and concentrations of secreted cytokine were assessed relative to standard curve. The mean +/- SD PPD-J specific (PPD-J induced cytokine concentration – unstimulated cytokine concentration) is shown for n = 6 (HAV-vaccinated) or n = 5 (Sham-vaccinated) animals including samples taken week -11, immediately prior prime vaccination; week -5, immediately prior boost vaccination; week 0, immediately prior to MAP challenge and up to 36 weeks post challenge. Significance between groups is indicated as * *P < *0.05, ** *P < *0.01.

**Figure 8:**
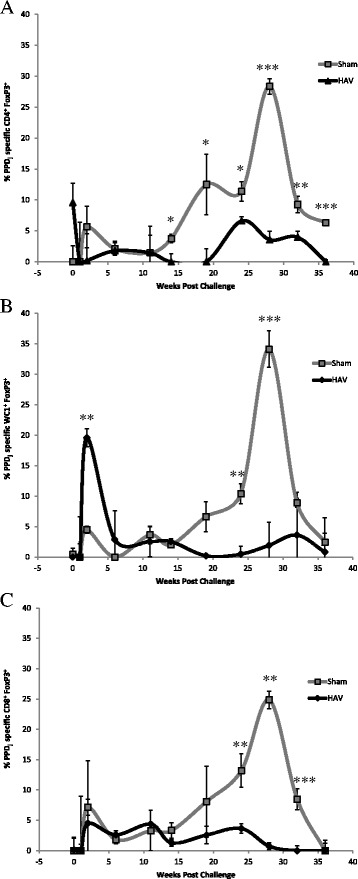
**FoxP3 expression by T cell sub-populations from HAV- and Sham-vaccinated animals.** PBMC from HAV-vaccinated (diamond) or Sham-vaccinated (square) calves were stimulated for 24 h with PPD-J or left unstimulated (control), then washed, fixed, permeablised and assessed for expression of **A**. CD4, **B**. WC1 and **C**. CD8. Cells were gated as live PBMC and the percentage of each cell population (CD4, CD8, WC1) expressing FoxP3 was calculated. Samples taken include week 0, immediately prior to MAP challenge and then up to 36 weeks post challenge. Significance between groups is indicated as * *P < *0.05, ** *P < *0.01, *** *P < *0.001.

Within a similar timeframe altered cytokine expression profiles were detected with significant differences between the HAV and Sham vaccinated groups (Figure 7). Levels of IL-1β peaked at week 11 (Figure 7A) followed a few weeks later by IL-10 (Figure 7B) with a significantly greater increase (*P < *0.05) in the secretion of both cytokines by PPD-J stimulated PBMC isolated from Sham vaccinated calves when compared to the HAV vaccinated group. In contrast an increase in PPD-J specific IFN-γ secretion was evident in the HAV vaccinated group at week 11 (Figure 1A) and antigen-specific expression of IFN-γ was significantly elevated in CD4^+^, CD8^+^ and WC1^+^ T cells from HAV vaccinated animals between 11 and 19 weeks (Figure 3A). No significant increases in IFN-γ were detected in Sham vaccinated animals in this time period. Within PBMC, alterations in the number of PPD-J stimulated cells expressing FoxP3 were evident from week 14 post-challenge with significant increases in CD4^+^FoxP3^+^ cells evident in the Sham vaccinated group at weeks 14 and 19 (*P* < 0.05; Figure 8A).

### Post challenge (20 - 38 weeks) and post-mortem

Towards the end of the challenge period the differences between HAV vaccinated and Sham vaccinated animals became more pronounced for a number of the measured parameters. The MAP killing capacity of the PBMC fraction returned in the Sham vaccinated group to levels similar to that of the HAV vaccinated group (Figure 6). The frequency of detection of MAP within PBMC increased in the Sham vaccinated group with 3/5 animals being consistently positive and all animals in this group testing positive at least once during this period (Figure 4). In contrast PBMC from 4/6 HAV vaccinated calves remained consistently negative and 2/6 only tested positive once within this period (Figure 4).

Antigen-specific cytokine levels decreased from week 20 onwards. At week 24 in the HAV vaccinated group significantly elevated levels of PPD-J specific IL-22 and raised levels of IL-17 were observed compared to the Sham vaccinated calves (Figure 5) which decreased along with antigen specific IL-1β and IL-10 over time (Figure 7). IL-10 levels remained significantly higher in the Sham vaccinated compared to the HAV vaccinated calves throughout the remainder of the study period.

The percentage of PPD-J specific IFN-γ expressing cells (Figure 3) and secreted IFN-γ (Figure 1) began to decrease in the HAV vaccinated group during this final stage whilst levels in the Sham vaccinated group remained low. A highly significant increase (*P < *0.001) in PPD-J specific FoxP3 expressing CD4^+^ (Figure 8A), WC1^+^ (Figure 8B) and CD8^+^ (Figure 8C) T cells was observed in the Sham vaccinated group compared to the HAV vaccinated animals from week 24 onwards (Figure 8).

**Figure 9:**
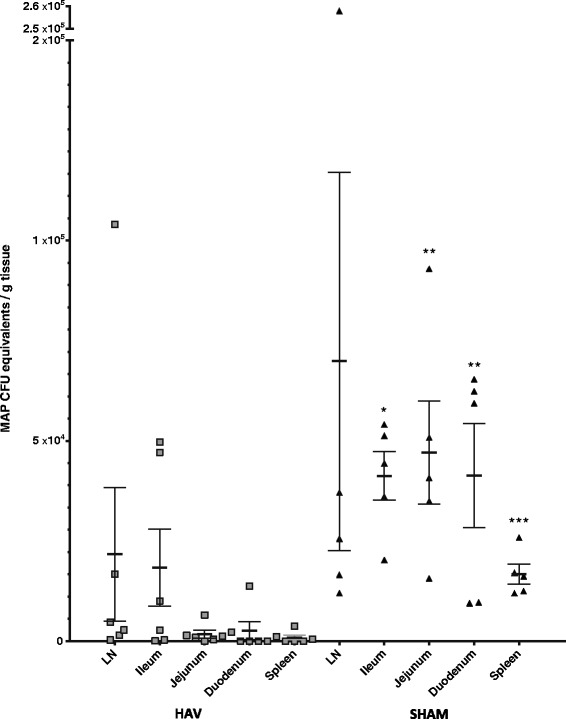
**MAP load in tissue 38 weeks post challenge.** Mean and SEM of genome equivalents determined by MAP specific IS900 qPCR (assuming 17 copies per organism) in weighed samples of various tissue sites (4 lymph node, 3 ileum, 6 jejunum, 2 duodenum, 1 spleen, per animal) from HAV vaccinated (triangles) and Sham vaccinated (squares) calves taken 38 weeks post MAP challenge. Each point represents an average of all samples taken from each tissue site in a single animal with individual sample values being derived from averages of duplicate qPCR performed on DNA extracted from each sample. Mann-Whitney U tests show Sham vaccinated animals had significantly greater loads than HAV vaccinated animals in duodenum (*P* = 0.003), jejunum (*P* = 0.009) and spleen (*P* = 0.002).

Standard tuberculin skin testing was carried out at week 36. All calves had similar skin reactivity to both PPD-A and PPD-B. The difference in PPD-B:PPD-A specific response was consistently < 1 mm indicating that none of the calves would be classified as TB reactors (see Additional file 1). At this time point DIVA testing using HAV specific antigens could still identify the HAV vaccinated from the Sham vaccinated calves (Figure 2). Examination of tissues taken post-mortem (38 weeks) revealed significant differences between the HAV vaccinated and Sham vaccinated groups. Measurement of the number of MAP present within tissues demonstrated significant reductions in load averages between HAV and Sham groups with samples obtained from duodenum (*P* = 0.003), jejunum (*P* = 0.009) and spleen (*P* = 0.002) (Figure 9). A significant decrease (*P* = 0.016) in overall total load was also observed when averages of all 5 sites were combined. All calves had at least one tissue sample positive for MAP indicating that whilst there was a significant degree of protection based on a significant reduction in bacterial load, sterilising immunity was not induced by HAV vaccination. All tissue samples from Sham vaccinated calves were positive for MAP. By contrast only 36/106 (34%) of all samples and 8/36 (17%) jejunum samples from the HAV vaccinated group were positive for MAP by qPCR. Nearly half (48%) of the total load present in HAV animals was located in mesenteric lymph node samples with 38% represented in one lymph node sample alone.

**Figure 10:**
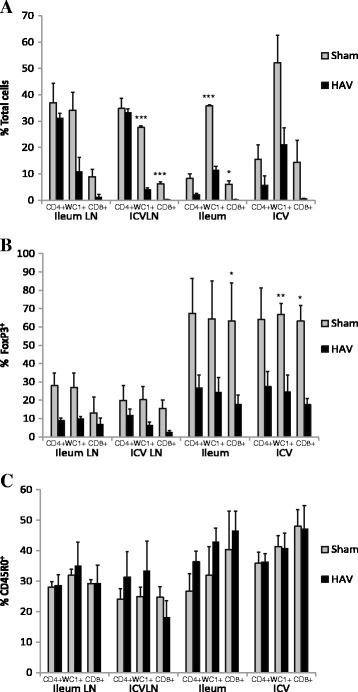
**FoxP3 and CD45RO cell populations in gut tissue 38 weeks post challenge.** Percentage of CD4^+^, WC1 ^+^, CD8 ^+^ cells in mucosal and lymph node tissue from ileal, ileocaecal valve sites **A**. total fraction; **B**. fraction expressing FoxP3; **C**. fraction expressing CD45RO from HAV vaccinated (black) or Sham vaccinated (grey) taken 38 weeks post challenge. Significance between groups is indicated as * *P < *0.05, ** *P < *0.01.

MAP cultured from two samples was shown to have the same genomic identity profile as the challenge strain (see Additional file 2). There were no obvious clinical manifestations or major macroscopic lesions at post mortem suggestive of progression towards clinical JD in any of the calves. Assessment of lymphoid cell populations within gut mucosal tissue from the ileum, ileocaecal valve region and associated lymph nodes showed larger populations of CD4^+^FoxP3^+^, CD8^+^FoxP3^+^ and WC1^+^FoxP3^+^ cells in the Sham vaccinated group compared to the HAV vaccinated group with the latter two reaching statistical significance (*P < *0.05; Figure 10). There was also trend towards an increased presence of IL-17 and IL-22 in lymph node tissue of the HAV vaccinated group but this did not reach significance (see Additional file 3).

## Discussion

Johne’s disease (JD) is a disease with economic significance in many dairy producing countries. Despite awareness of the problem and long term implementation of extensive control policies, MAP prevalence in domestic livestock worldwide has been rapidly increasing, particularly in dairy cattle. Vaccination is the most cost effective disease control measure but current whole cell killed JD vaccines have limited efficacy and are incompatible with diagnosis of MAP infection. Notably these also interfere with bovine tuberculosis tuberculin skin tests. In previous studies using a mouse model we have shown that a prime-boost viral delivery regimen of early entry MAP specific antigens (HAV vaccine) showed significant protection and efficacy in prevention of colonisation [23].

In this study we have applied this approach to cattle and shown that prime-boost HAV vaccination prior to MAP challenge offered a high degree of protection relative to a Sham vaccinated challenged group. We report protective capacity as an ability of vaccination to significantly reduce bacterial numbers in peripheral blood, gut tissues and in faeces as this is likely to impact significantly on disease progression and transmission.

Significant infection of Sham vaccinated calves with MAP was shown herein. MAP could be detected in 100% of tissue samples at 38 weeks post challenge at high numbers (up to 5 logs of MAP load per gram of tissue) and shedding was detected at least once in faecal samples from 3/5 Sham vaccinated challenged animals. Contrastingly, all faecal samples and at least one tissue sampling site from 4/6 HAV vaccinated calves tested negative (less than 100 organisms per gram) for the presence of MAP.

This reduction in faecal shedding would have a major impact on disease control strategies and would contribute to minimising animal to animal spread of MAP infection. Examination of a range of immunological parameters suggested that these occurred in distinct phases related to the infectious load of MAP and these differed between HAV and Sham vaccinated animals. During the initial phase HAV vaccination significantly primed and boosted antigen specific CD4^+^, CD8^+^ but not WC1^+^ IFN-γ producing T-cell populations. No adverse events and no excretion of vaccine were detected one week and up to 43 weeks post vaccination indicating that, as in other studies, MVA and adenoviral delivery systems are well tolerated in cattle. The DIVA peptide pool used, despite being derived from MAP specific epitopes, showed reactivity only with HAV vaccinated animals and was significantly raised throughout the experiment. Reactivity was not increased after MAP challenge and importantly no response to the HAV specific peptides was observed in in Sham vaccinated animals post-MAP challenge suggesting that these epitopes are not recognised during early natural MAP infection in cattle. In addition none of the cattle in the current study tested positive in the tuberculin skin test used for diagnosis of bovine TB. This is important as it is essential not only to be able to distinguish HAV vaccinated from MAP infected cattle in DIVA tests but also to be able to identify these from cattle infected with *M. bovis*. This will be of particular importance in countries with ongoing bovine TB control programmes such as the UK. All animals were challenged orally with MAP, which resulted in rapid and efficient uptake as demonstrated by the high degree of transient MAP bacteraemia in the peripheral blood observed at week 1 post-challenge. Some early passive shedding was seen along with pro-inflammatory Th1 immunological responses and T cell proliferative responses characteristic of established MAP infection [34]. Importantly in the HAV vaccinated group we observed a more rapid and significantly higher expression of IFN-γ compared with the Sham-vaccinated cattle likely to indicate early Th1 polarisation. This enhanced secretion of IFN-γ in HAV vaccinated calves could activate macrophages for enhanced cytotoxicity. Indeed in this study we demonstrated that macrophages from HAV vaccinated cattle had, at early time points post-infection, significantly greater capacity to kill MAP compared to PBMC taken from Sham vaccinated animals. This may be a crucial early event determining the eventual outcome of infection. Interestingly, early post-challenge there was a dramatic loss in the capacity of PBMC derived macrophages to kill MAP that was only observed in the Sham vaccinated animals and not the HAV vaccinated calves. This may reflect the capacity of MAP to actively evade killing within macrophages and to alter their lytic capacity such that in infected calves the normal killing capacity is significantly reduced. An alternative, but not exclusive explanation is that HAV vaccination induced immune responses (including IFNγ release and T cell activation) activate macrophages for enhanced killing. Our studies have not defined which parameters are required for the maintenance of killing capacity observed in HAV vaccinated cattle but this will be an important aspect to dissect in future studies.

After 2 weeks the number of animals with MAP detectable within PBMC declined; this was evident in both the HAV vaccinated and Sham vaccinated animals. For the remainder of the experiment only one animal in the HAV vaccinated group continued to have persistent MAP present in the PBMC fraction. However, in the Sham vaccinated animals the reduction observed at 2 weeks was transient and persistent bacteraemia returned, consistent with the hypothesis that vaccination significantly affects the capacity of the host to control MAP. The transient reduction in the number of MAP present within the blood of Sham vaccinated animals may correspond to a translocation of MAP to tissues where they begin to divide before again populating the blood at later stages of infection. By contrast the ongoing immune response in the HAV vaccinated calves is likely to contribute to the level of MAP proliferation and/or survival. Rapid dissemination of MAP post infection and consistent bacteraemia has been demonstrated in several animal models for at least 72 hours post challenge [35]. Long term bacteraemia in naturally infected animals has been linked with progression towards disease, particularly that of the multibacillary type [36]. Thus, early control of peripheral bacteraemia by vaccination may be critical for long term protection from disease.

Small increases in IL-17 were also detected in HAV vaccinated cattle early post-infection, however due to a lack of antibodies for detection of intra-cytoplasmic IL-17 in cattle, we were not able to determine the cellular source of IL-17 herein. Both IFN-γ and IL-17 have been implicated as protective cytokines induced by MAP vaccination in previous studies [37]. Conversely, the cytokine response in the Sham vaccinated animals at early time points were dominated by IL-1β and IL-10 with little induction of IFN-γ. Similar profiles have previously been associated with late stage intracellular processing of mycobacteria [38] and progression of MAP infection [39]. The source, timing and magnitude of IL-10 production can be a major determinant on disease outcome [40] and the induction of an IL-10 response in animals with significant MAP burden (i.e. the Sham vaccinated calves) is an indicator of immune regulatory imbalance which could facilitate intracellular mycobacterial survival [41]. A number of studies have shown the source of IL-10 from MAP infected cattle to be largely CD4^+^ T cells, although monocytes may also be involved. In vitro upregulation of expression of IL-10 is a major response mechanism of bovine macrophages infected with MAP and is associated with reduced IFN-γ secretion and immune evasion. In bovine intestinal tissues early post-infection, MAP induces anti-inflammatory genes such as IL-10 [42] associated with increased intracellular survival. Furthermore we have recently demonstrated that knockdown of IL-10 by siRNA significantly inhibits intracellular survival of BCG indicating a key role for IL-10 in enabling mycobacterial growth and persistence in macrophages (Professor Liz Glass, personal communication to J Hope).

Up-regulated IL-1β has been described in the tissues of animals affected by JD [40], the expression of which appeared to correlate with inflammation. In an epithelial cell line-bone marrow-derived macrophage (bMDM) co-culture model, MAP invasion of the epithelial cells induced up-regulation of IL-1β, leading to the transmigration of the bMDM [43]. This may be a mechanism whereby MAP promotes its own uptake and intracellular survival. Since IL-1β (along with IL-23/IL-17) is regulated by autophagy, interference with expression of these cytokines could also indicate that MAP is directly subverting this pathway to promote its survival within macrophages enabling growth and establishment within the host. This is in line with the decreased capacity of PBMC to kill MAP that we observed in the infected Sham vaccinated calves.

In the final phase of this study (> 19 weeks) MAP bacteraemia steadily increased and persisted in the Sham vaccinated calves but stayed low in the HAV vaccinated group. The final MAP load in tissues of HAV vaccinated animals at 38 weeks was significantly reduced in gut and lymphoid tissues with most (73%) gut mucosal tissue samples testing MAP negative and the majority (55%) of detectable MAP organisms were located in lymph nodes. Faecal shedding was not an expected outcome measure in this model due to the low challenge dose and short study duration post challenge [30]. However abrogation of faecal shedding is a major requirement for an effective MAP vaccine [9] so it was interesting to note that all HAV vaccinated animals were negative for MAP in faecal samples collected throughout the experiment whilst intermittent faecal positives were detected in the Sham vaccinated group.

Additionally in this final phase, the Sham vaccinated group developed increased proportions of PPD-J reactive FoxP3^+^ T cell populations which were also evident in gut tissues taken at the end of the study period. This may be consistent with the development of a regulatory population of T cells, although we have not demonstrated herein that these cells display such functions. Reduced CD4+ effector T cell capacity and the development of regulatory T cell populations has been reported to correlate with disease progression in other studies of MAP infection in cattle, consistent with our observations [44,45]. Interestingly we found significant alterations in the proportion of WC1^+^ γδ TCR^+^ T cells present within the tissues of HAV vaccinated compared to Sham vaccinated cattle. A significant proportion of WC1^+^ γδ TCR^+^ T cells are regulatory, expressing high levels of IL-10 [46,47] which could contribute to the continued ability of MAP to proliferate within the tissue. A caveat to the observations and interpretations of our data is the relatively short duration of the experimental infection model used herein. More extensive studies in a long term model or in a field study where natural exposure to MAP occurs will be required to confirm whether we can reproducibly eliminate faecal shedding. This would represent a major advance in disease control as breaking the transmission cycle would have a significant impact on the incidence and spread of disease within and between herds. Such longitudinal studies in large cohorts of MAP exposed cattle would enable us to define the impact of HAV vaccination not only on transmission but also on disease progression. This is an essential next step in confirming the protective efficacy of the HAV vaccine.

The majority of recent MAP vaccination strategies have relied on MAP whole cell formulations to effect nonspecific multi-antigen delivery, thus it is difficult to make detailed comparisons between our novel specific multi-epitope viral delivery and other vaccine regimens. However, studies have shown that protective immunity is associated with high IFN-γ levels and increased Th-17 related responses [22]. There is evidence that the failure of whole cell vaccines to eliminate MAP shedding in faeces may be due partly to interference from non-specific immune regulators present in the mycobacterial cell wall that can deflect appropriate induction of Th1 responses critical for disease resolution [34] and reduce antigen presentation [48]. By contrast HAV vaccination appeared to induce appropriate immune bias, enhanced MAP-specific killing and eliminated MAP shedding.

A long term challenge will be the implementation of HAV vaccination in the field since the strategy that we have assessed involves prime-boost with viral, genetically modified, vectors rather than a single subunit vaccination. Assessment of the MAP-specific antigens contained within the HAV vaccine in combination with adjuvants or other delivery systems will be important, as will determination of the duration of immunity induced by vaccination and the long term impact of vaccination on MAP infection and Johne’s disease in the face of potential high-level exposure in heavily affected herds.

In conclusion we have shown that prime-boost viral delivery of MAP antigens to young calves was well tolerated, vaccine was not excreted and vaccination was able to prime a range of cell mediated immune responses which may correlate with the induction of protective immunity. We have shown significant efficacy of HAV vaccination of young calves to reduce the tissue burden of MAP associated with abrogated faecal shedding of MAP. These features, alongside a clear capacity to differentiate vaccinated from infected animals by a novel DIVA test, lack of tuberculin cross reactivity and definition of immunological parameters associated with varied stages post-infection highlight the promise of the HAV vaccine for the improved control of MAP infection in cattle.

## Additional files

Additional file 1:
**Tuberculin testing of Sham vaccinated (grey) and HAV vaccinated (black) animals 35 weeks post MAP challenge.** Table showing skin thickness measurements at separate PPA-A, PPD-B inoculum sites pre and 72 h post inoculation. A standard positive tuberculin test requires > 5 mm difference between PPD-B and PPD-A at 72 h. A standard positive tuberculin test requires > 5 mm difference. No significant difference between groups was demonstrated (*P* = 0.523) [49]. Additional file 2:
**MIRU-VNTR typing of MAP isolates.** Gel files showing specific MIRU-VNTR PCR products profiles comparing Control K10 reference strain, challenge strain MAP R0808 and a MAP isolate from faeces of Sham vaccinated animal obtained at 36 weeks. Additional file 3:
**IL-17 and IL-22 in tissue from Sham vaccinated and HAV vaccinated animals 36 weeks post MAP challenge.** Bar graphs showing fold increases relative to GAPDH in expression of cytokines Graph A. IL-17 and Graph B. IL-22 in mucosal and lymph node tissue from ileal, ileocaecal valve sites obtained from HAV vaccinated (black) or Sham vaccinated (grey) calves obtained 38 weeks post challenge. There were no significance between groups (*P* > 0.05).
