# Supplementary material for: Immunity, safety and protection of an Adenovirus 5 prime - Modified Vaccinia virus Ankara boost subunit vaccine against Mycobacterium avium subspecies paratuberculosis infection in calves
Source: Vet Res. 2014 Oct 29;45(1):112. doi: 10.1186/s13567-014-0112-9 (PMC4258034; doi:10.1186/s13567-014-0112-9)
Supplement: Additional file 1: — Tuberculin testing of Sham vaccinated (grey) and HAV vaccinated (black) animals 35 weeks post MAP challenge. Table showing skin thickness measurements at separate PPA-A, PPD-B inoculum sites pre and 72 h post inoculation. A standard positive tuberculin test requires > 5 mm difference between PPD-B and PPD-A at 72 h. A standard positive tuberculin test requires > 5 mm difference. No significant difference between groups was demonstrated (P = 0.523) [49]. [file 13567_2014_112_MOESM1_ESM.pptx]

## Slide 1
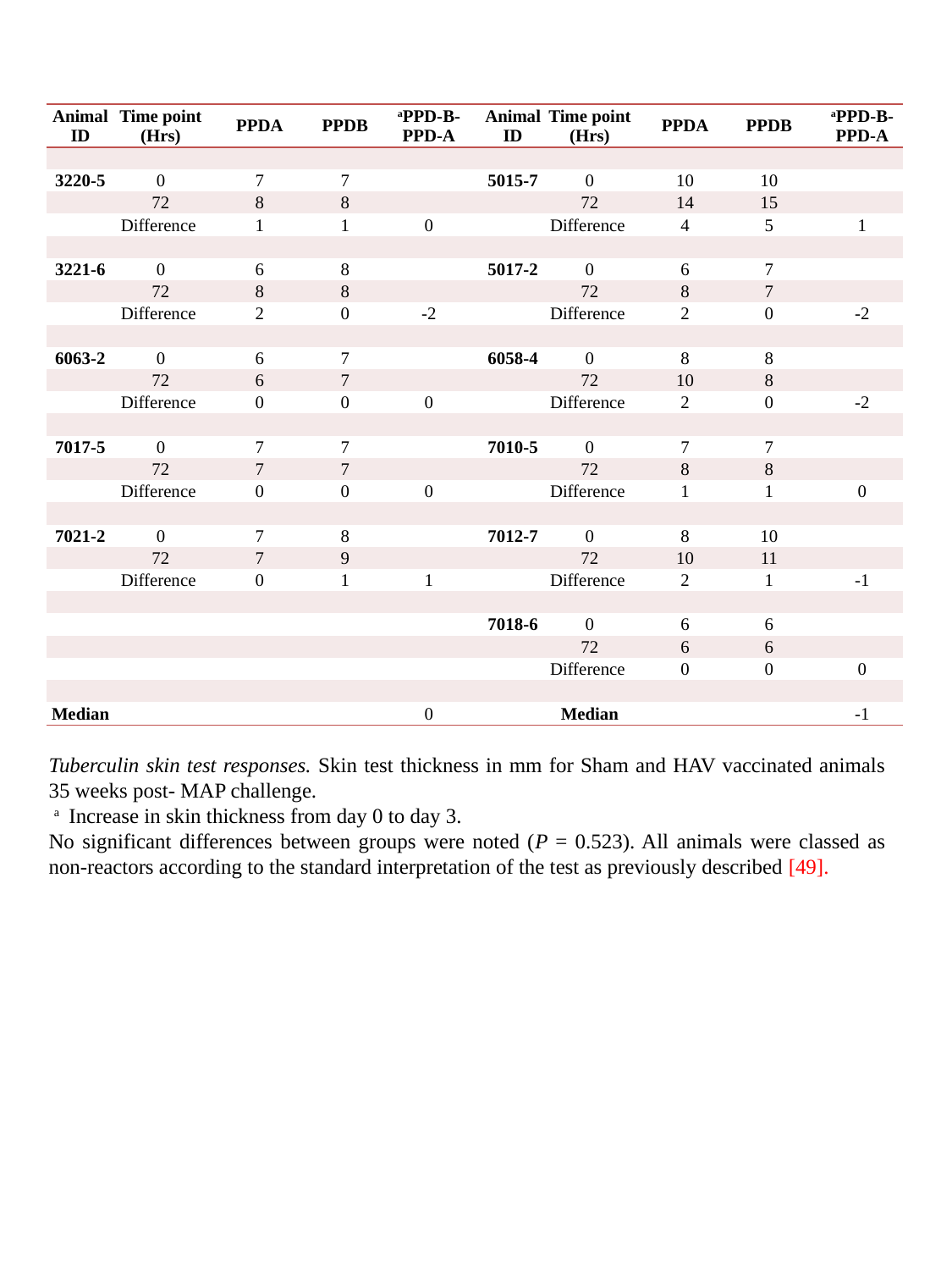

| Animal ID | Time point (Hrs) | | PPDA | | PPDB | | aPPD-B-PPD-A | | Animal ID | Time point (Hrs) | | PPDA | | PPDB | | aPPD-B-PPD-A |
| --- | --- | --- | --- | --- | --- | --- | --- | --- | --- | --- | --- | --- | --- | --- | --- | --- |
| | | | | | | | | | | | | | | | | |
| 3220-5 | 0 | | 7 | | 7 | | | | 5015-7 | 0 | | 10 | | 10 | | |
| | 72 | | 8 | | 8 | | | | | 72 | | 14 | | 15 | | |
| | Difference | | 1 | | 1 | | 0 | | | Difference | | 4 | | 5 | | 1 |
| | | | | | | | | | | | | | | | | |
| 3221-6 | 0 | | 6 | | 8 | | | | 5017-2 | 0 | | 6 | | 7 | | |
| | 72 | | 8 | | 8 | | | | | 72 | | 8 | | 7 | | |
| | Difference | | 2 | | 0 | | -2 | | | Difference | | 2 | | 0 | | -2 |
| | | | | | | | | | | | | | | | | |
| 6063-2 | 0 | | 6 | | 7 | | | | 6058-4 | 0 | | 8 | | 8 | | |
| | 72 | | 6 | | 7 | | | | | 72 | | 10 | | 8 | | |
| | Difference | | 0 | | 0 | | 0 | | | Difference | | 2 | | 0 | | -2 |
| | | | | | | | | | | | | | | | | |
| 7017-5 | 0 | | 7 | | 7 | | | | 7010-5 | 0 | | 7 | | 7 | | |
| | 72 | | 7 | | 7 | | | | | 72 | | 8 | | 8 | | |
| | Difference | | 0 | | 0 | | 0 | | | Difference | | 1 | | 1 | | 0 |
| | | | | | | | | | | | | | | | | |
| 7021-2 | 0 | | 7 | | 8 | | | | 7012-7 | 0 | | 8 | | 10 | | |
| | 72 | | 7 | | 9 | | | | | 72 | | 10 | | 11 | | |
| | Difference | | 0 | | 1 | | 1 | | | Difference | | 2 | | 1 | | -1 |
| | | | | | | | | | | | | | | | | |
| | | | | | | | | | 7018-6 | 0 | | 6 | | 6 | | |
| | | | | | | | | | | 72 | | 6 | | 6 | | |
| | | | | | | | | | | Difference | | 0 | | 0 | | 0 |
| | | | | | | | | | | | | | | | | |
| Median | | | | | | | 0 | | | Median | | | | | | -1 |
Tuberculin skin test responses. Skin test thickness in mm for Sham and HAV vaccinated animals 35 weeks post- MAP challenge.
 a Increase in skin thickness from day 0 to day 3.
No significant differences between groups were noted (P = 0.523). All animals were classed as non-reactors according to the standard interpretation of the test as previously described [49].
