# Supplementary material for: Immunity, safety and protection of an Adenovirus 5 prime - Modified Vaccinia virus Ankara boost subunit vaccine against Mycobacterium avium subspecies paratuberculosis infection in calves
Source: Vet Res. 2014 Oct 29;45(1):112. doi: 10.1186/s13567-014-0112-9 (PMC4258034; doi:10.1186/s13567-014-0112-9)
Supplement: Additional file 2: — MIRU-VNTR typing of MAP isolates. Gel files showing specific MIRU-VNTR PCR products profiles comparing Control K10 reference strain, challenge strain MAP R0808 and a MAP isolate from faeces of Sham vaccinated animal obtained at 36 weeks. [file 13567_2014_112_MOESM2_ESM.pptx]

## Slide 1
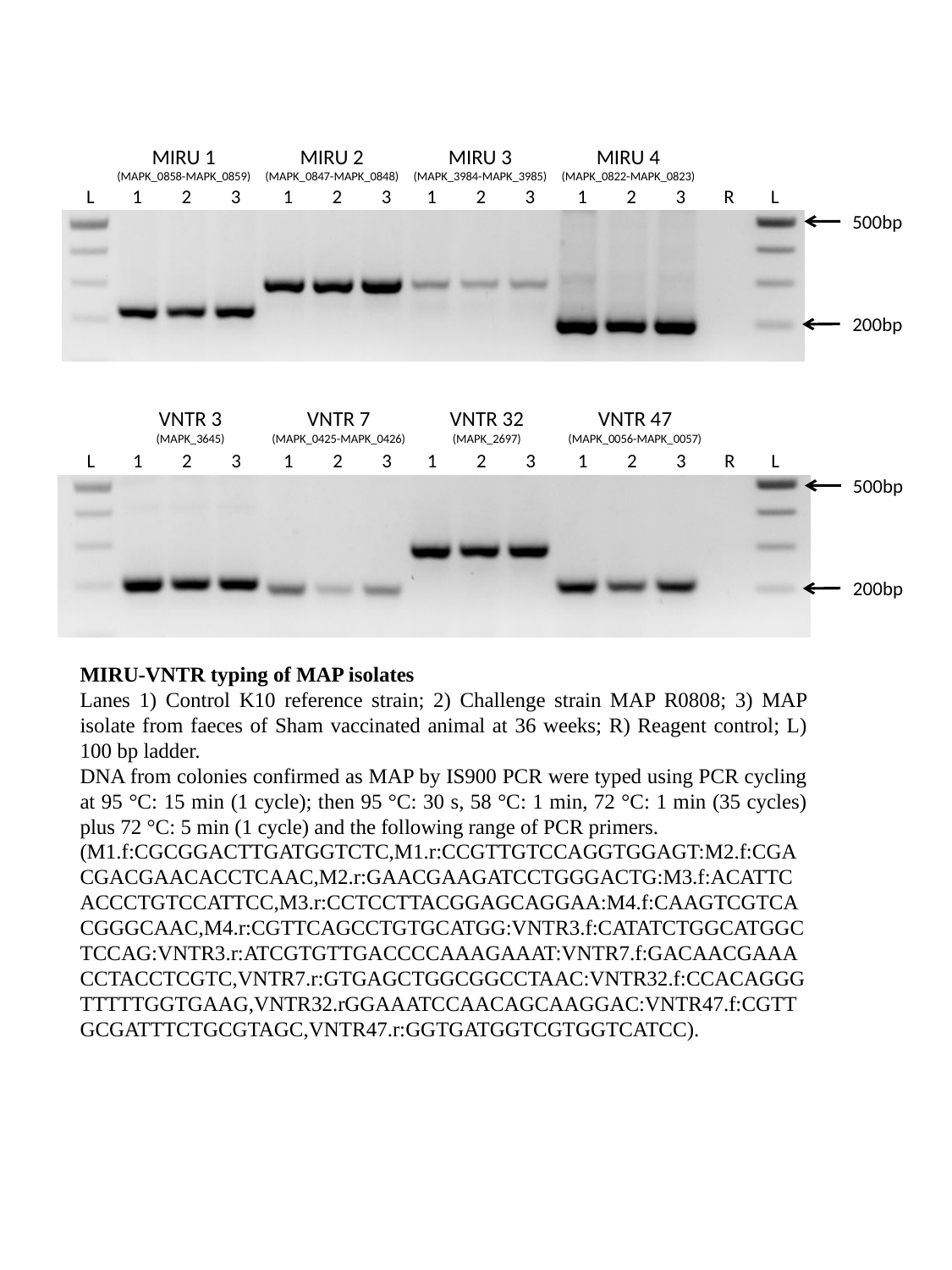

MIRU 1
(MAPK_0858-MAPK_0859)
MIRU 2
(MAPK_0847-MAPK_0848)
MIRU 3
(MAPK_3984-MAPK_3985)
MIRU 4
(MAPK_0822-MAPK_0823)
L
1
2
3
1
2
3
1
2
3
1
2
3
R
L
500bp
200bp
VNTR 3
(MAPK_3645)
VNTR 7
(MAPK_0425-MAPK_0426)
VNTR 32
(MAPK_2697)
VNTR 47
(MAPK_0056-MAPK_0057)
L
1
2
3
1
2
3
1
2
3
1
2
3
R
L
500bp
200bp
MIRU-VNTR typing of MAP isolates
Lanes 1) Control K10 reference strain; 2) Challenge strain MAP R0808; 3) MAP isolate from faeces of Sham vaccinated animal at 36 weeks; R) Reagent control; L) 100 bp ladder.
DNA from colonies confirmed as MAP by IS900 PCR were typed using PCR cycling at 95 °C: 15 min (1 cycle); then 95 °C: 30 s, 58 °C: 1 min, 72 °C: 1 min (35 cycles) plus 72 °C: 5 min (1 cycle) and the following range of PCR primers.
(M1.f:CGCGGACTTGATGGTCTC,M1.r:CCGTTGTCCAGGTGGAGT:M2.f:CGACGACGAACACCTCAAC,M2.r:GAACGAAGATCCTGGGACTG:M3.f:ACATTCACCCTGTCCATTCC,M3.r:CCTCCTTACGGAGCAGGAA:M4.f:CAAGTCGTCACGGGCAAC,M4.r:CGTTCAGCCTGTGCATGG:VNTR3.f:CATATCTGGCATGGCTCCAG:VNTR3.r:ATCGTGTTGACCCCAAAGAAAT:VNTR7.f:GACAACGAAACCTACCTCGTC,VNTR7.r:GTGAGCTGGCGGCCTAAC:VNTR32.f:CCACAGGGTTTTTGGTGAAG,VNTR32.rGGAAATCCAACAGCAAGGAC:VNTR47.f:CGTTGCGATTTCTGCGTAGC,VNTR47.r:GGTGATGGTCGTGGTCATCC).
