# Supplementary material for: Immunity, safety and protection of an Adenovirus 5 prime - Modified Vaccinia virus Ankara boost subunit vaccine against Mycobacterium avium subspecies paratuberculosis infection in calves
Source: Vet Res. 2014 Oct 29;45(1):112. doi: 10.1186/s13567-014-0112-9 (PMC4258034; doi:10.1186/s13567-014-0112-9)
Supplement: Additional file 3: — IL-17 and IL-22 in tissue from Sham vaccinated and HAV vaccinated animals 36 weeks post MAP challenge. Bar graphs showing fold increases relative to GAPDH in expression of cytokines Graph A. IL-17 and Graph B. IL-22 in mucosal and lymph node tissue from ileal, ileocaecal valve sites obtained from HAV vaccinated (black) or Sham vaccinated (grey) calves obtained 38 weeks post challenge. There were no significance between groups (P > 0.05). [file 13567_2014_112_MOESM3_ESM.pptx]

## Slide 1
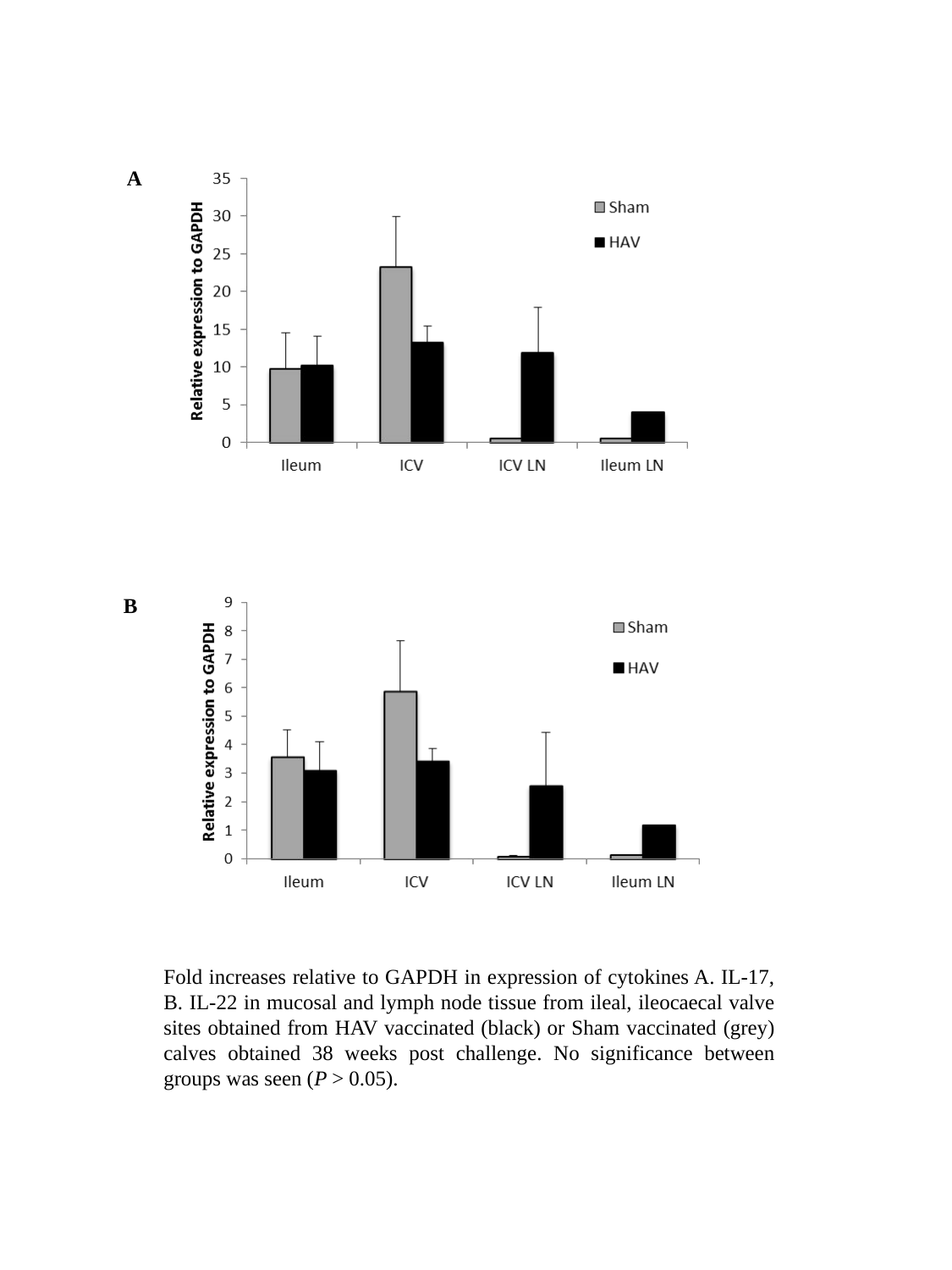

A
B
Fold increases relative to GAPDH in expression of cytokines A. IL-17, B. IL-22 in mucosal and lymph node tissue from ileal, ileocaecal valve sites obtained from HAV vaccinated (black) or Sham vaccinated (grey) calves obtained 38 weeks post challenge. No significance between groups was seen (P > 0.05).
